# Electrically evoked late latency response using single electrode stimulation and its relation to speech perception among paediatric cochlear implant users

**DOI:** 10.3389/fnhum.2024.1441854

**Published:** 2024-09-13

**Authors:** Palani Saravanan, Neelamegarajan Devi, Chinnaraj Geetha

**Affiliations:** ^1^Department of Audiology, Centre for Hearing Sciences (CHS), All India Institute of Speech and Hearing (AIISH), Mysuru, India; ^2^Department of Audiology, All India Institute of Speech and Hearing (AIISH), Mysuru, India

**Keywords:** electrically evoked late latency response, cortical auditory evoked potentials, cochlear implant, single electrode stimulation, P1-N1 response, speech perception

## Abstract

**Introduction:**

Aided auditory late latency response (LLR) serves as an objective tool for evaluating auditory cortical maturation following cochlear implantation in children. While aided LLR is commonly measured using sound-field acoustic stimulation, recording electrically evoked LLR (eLLR) offer distinct advantages, such as improved stimulus control and the capability for single electrode stimulation. Hence, the study aimed to compare eLLR responses with single electrode stimulation in the apical, middle, and basal regions and to evaluate their relationship with speech perception in paediatric cochlear implant (CI) recipients.

**Method:**

eLLR responses with single electrode stimulation were measured in 27 paediatric unilateral CI users with an active recording electrode placed at Cz. The stimuli consisted of 36 msec biphasic pulse trains presented across three electrode sites (apical-E20, middle-E11, and basal-E03). eLLR responses were compared across these electrode sites, and the relationship between speech recognition scores in quiet and age at implantation with eLLR components was evaluated.

**Results:**

eLLR responses were detected in 77 out of 81 tested electrodes of all participants combined (27 for apical, 26 for middle, and 24 for basal stimulation). There were no significant differences in P1, N1 latencies and P1 amplitude across electrode site. However, significantly larger N1 and P1-N1 amplitudes were observed for apical stimulations compared to basal stimulations. No differences in N1 amplitude were found between middle and apical stimulations, and the P1-N1 amplitude was significantly larger for middle compared to basal electrode stimulation, with no difference between the apical and middle electrodes stimulation. A moderate positive correlation was present between speech recognition scores in quiet and both N1, P1-N1 amplitudes for apical stimulation. Age at implantation was negatively correlated with N1 amplitude for the apical and P1-N1 amplitude for basal stimulation.

**Discussion:**

eLLR responses could be elicited in majority of paediatric CI users across electrode sites. Variations in eLLR responses across electrode sites suggest disparities in auditory cortical maturation. The findings underscore the significance of the N1 biomarker in evaluating higher-order auditory cortical development. Therefore, utilizing eLLR with single electrode stimulation may serve as a valuable tool for assessing post-cochlear implantation maturational changes in paediatric populations.

## 1 Introduction

More than one million individuals worldwide have cochlear implants (CI) (Zeng, 2022). Wilson et al. (2011) highlighted that the CI is the most successful neural prosthesis developed so far. The CI significantly enhances the quality of life for people with severe to profound hearing loss, both in children and adults (Entwisle et al., 2018; Sousa et al., 2018; Teagle et al., 2019). Despite this success, there are notable disparities in CI outcomes, which remain a key clinical concern in both paediatric (Niparko, 2010; Barnard et al., 2015) and adult populations (Blamey et al., 2015; Holden et al., 2013).

Speech perception with a CI is influenced by how the auditory signal is processed in the central auditory system. Congenital hearing loss and delayed implantation can have a significant impact on typical auditory cortical maturation, leading to substantial effects on CI outcomes (Kral and Sharma, 2012; Sharma et al., 2002a, 2005). A crucial aspect of clinical practice in audiology is the evaluation of the central auditory system performance in CI users through auditory late latency response (LLR) measurement (Gordon et al., 2005, 2008).

The LLR is a non-invasive electroencephalographic (EEG) technique to assess the auditory cortical maturation in children with hearing loss (Sharma et al., 2002a, 2002b; Sharma et al., 2002c). The obligatory LLR comprises three components: P1, N1, and P2. In a mature auditory system, all the three peaks are evident. However, in young children, the LLR is primarily characterized by the prominent positive peak (P1) occurring between 100 and 300 milliseconds (msec) after stimulus onset (Eggermont et al., 1997; Sharma et al., 2002a). As children age, the latency of P1 decreases, and the negative peak (N1) emerges, which distinguishes the positive peak into P1 and P2 (Sharma et al., 2015a). The obligatory nature of the P1 and N1 response, which does not necessitate a child’s attention to the stimulus, makes them suitable for hearing assessment and for evaluating the cortical maturation of primary and higher-order cortical structures objectively in children with hearing loss (Kral and Sharma, 2012; Sharma et al., 2015a).

In paediatric CI users, the P1 response in the aided late latency responses (LLR) can serve as a biomarker for evaluating auditory cortical maturation post-implantation (Gordon et al., 2008; Purdy and Gardner-Berry, 2009; Sharma et al., 2015a, 2016). The P1 responses originate from the primary auditory cortex and thalamic regions (Eggermont and Ponton, 2003; Kral and Eggermont, 2007; Ponton et al., 2000). Studies indicate that normal P1 latency and morphology are more common in children implanted before the age of 3.5 years than those implanted after 7 years. Among children implanted between 3.5 and 7 years, 50% exhibited normal P1 responses (Sharma et al., 2002b; Sharma and Dorman, 2006). Researchers concluded that there exists a critical period (< 3.5 years) for the proper development of the central auditory nervous system, during which auditory system stimulation should commence.

Additionally, the appearance of the N1 peak in auditory cortical potentials has also been associated with the development of advanced auditory perceptual skills in CI users (Sharma et al., 2015a), such as speech perception in noisy environments and understanding degraded speech (Eggermont and Ponton, 2003; Ponton et al., 2000). The N1 component of the LLR originates from higher-order auditory cortex, reflecting long-term auditory cortical maturation in children with CI (Sharma et al., 2015a). A study involving 80 children with CI, it was found that 71% of early-implanted children (<3.5 years) exhibited N1 responses, while only 30% of children in the mid-implanted group (3.5–7 years) showed N1 responses, and none of the children in the late-implanted group displayed N1 responses (Sharma et al., 2015b). Therefore, N1 response could serve as a valuable tool for evaluating the maturation of higher-order cortical structures in children using CI.

Post-cochlear implantation, aided LLR are typically measured using sound-field acoustic presentation of stimuli (Cardon and Sharma, 2013; Dorman et al., 2007; Jeong et al., 2018; Kosaner et al., 2018; Sharma et al., 2002a; Sharma et al., 2005; Távora-Vieira et al., 2022a; Távora-Vieira et al., 2022b). The most frequently used acoustic stimuli for LLR recording include frequency-specific tone bursts and short speech syllables like /d/, /b/, /t/, /g/, /m/, etc. (Kosaner et al., 2018; Sharma et al., 2002a; Távora-Vieira et al., 2021). In recent years, it has been observed that direct electrical stimulation of intracochlear electrodes to record electrically evoked LLR (eLLR) offers several advantages over sound-field stimulation. Direct stimulation allows for better control over stimulus characteristics such as timing, amplitude and the stimulation location within the cochlea. This method circumvents the effects of external microphones, pre-processing algorithms, filter banks, and the users’ map levels, which can significantly modify the signal (Callejón-Leblic et al., 2023; Liebscher et al., 2018). Additionally, direct stimulation prevents the activation of multiple electrodes, leading to a more precise stimulation pattern and reducing the influence of physical factors like head movement and room acoustics (Távora-Vieira et al., 2021; Távora-Vieira et al., 2022a; Távora-Vieira et al., 2022b).

The parameters commonly used for CI stimulation can effectively elicit the eLLR response (Callejón-Leblic et al., 2023). While recent studies have explored eLLR recording in adult CI users (Callejón-Leblic et al., 2023; Kranick et al., 2021; Liebscher et al., 2018; Visram et al., 2015), research in the paediatric population remains limited. Gordon et al. (2005) identified three types of eLLR responses in paediatric CI users. Type 1 responses were characterized by a prominent positive peak, type 2 responses featured a prominent positive peak with slightly delayed latency, and type 3 responses showed a prominent negative peak followed by a positive peak. Children displaying type 3 responses exhibited poorer speech perception abilities compared to those with the typical type 1 and type 2 responses.

Furthermore, the processing of a signal transmitted to the central auditory nervous system by a CI may differ significantly between individuals, as the ability of users to adapt to novel neural patterns can vary widely (Abbas and Brown, 2015). These individual differences and the maturational changes of the central mechanisms after CI stimulation could be objectively evaluated by eLLR (Abbas and Brown, 2015; Gordon et al., 2005). Further, recording eLLR with individual electrode stimulation allows for measuring variations in central processing across different electrodes. Therefore, eLLR recorded with single-electrode stimulation can be a valuable measure to assess central processing following cochlear implantation. However, the functional response to electrical stimulation varies significantly from one site to another along the electrode array, and these patterns of implant function differ among individuals (Pfingst et al., 2015). Thus, recording eLLR at multiple electrodes is essential. Sequential stimulation of multiple electrodes along the array is crucial for perceiving complex signals, such as speech, which consists of different frequencies (Pfingst et al., 2015). Recording and comparing eLLR across different electrode sites aids in objectively evaluating auditory cortical areas at various frequency ranges. Assessing the relationship between eLLR recorded at multiple electrode sites post-cochlear implantation provides insights into whether there is parallel cortical maturation across different cortical regions.

Most studies have conducted eLLR assessments using direct intra-cochlear electrode stimulation in adults (Callejón-Leblic et al., 2023; Kranick et al., 2021; Liebscher et al., 2018; Tavora-Vieira and Ffoulkes, 2023). Liebscher et al. (2018) found a significant positive correlation between the N1 amplitude of eLLR and speech perception performance in adult CI users. The eLLR responses were comparable, reliable and had a good correlation with acoustically evoked LLR (Távora-Vieira et al., 2021). Understanding variations in eLLR across electrode locations and their relationship to speech perception in paediatric populations is crucial. Hence, the present study aimed to assess the impact of electrode locations on eLLR responses and their relationship with speech perception abilities among paediatric CI users.

## 2 Materials and methods

### 2.1 Participants

The study involved 27 paediatric unilateral CI recipients, consisting of 15 males and 12 females. The average age during testing was 5.86 years, with a range from 3.25 to 8 years and a standard deviation of 0.81 years. All participants had received the CI422 implant with a lateral wall array (Cochlear Nucleus implants) and had been using the CI for at least 1 year. The average age at implantation was 4.78 years (standard deviation = 0.82 years). The average duration of CI use was 1.06 years (standard deviation = 0.15 years).

The children were diagnosed with bilateral severe to profound congenital hearing loss and underwent unilateral cochlear implantation under a state government health scheme. All the participants had congenital onset of hearing loss, and exact cause for the hearing loss is unknown. Nineteen children received implants in the right ear, and eight received them in the left ear based on the pre-implant radiological evaluations. All participants had a normal cochlea and auditory nerves in the implanted ear. A fully inserted electrode array confirmed by intraoperative neural response telemetry measures and post-operative x-ray. All participants wore the CI during waking hours and used a digital behind-the-ear hearing aid in the non-implanted ear. Their aided sound-field thresholds with the CI ranged from 20 to 35 dB HL across frequencies of 250 to 8,000 Hz. The children received standard care, including CI programming and auditory verbal therapy, for one year. None of the participants had associated behavioural, cognitive, or neurological disorders. The demographic characteristics of the participants are summarized in Table 1.

**Table 1:** Demographic information of the participants of the present study.

Participant	Sex	Ear	Congenital/Acquired	Internal device	Speech processor	Age at implantation (years)	Age at testing (years)	Time in use (years)	Corrected speech recognition scores (%)
S1	M	Right	Congenital	CI422	CP802	4.92	5.92	1.00	36.00
S2	F	Right	Congenital	CI422	CP802	5.5	6.50	1.00	94.67
S3	M	Left	Congenital	CI422	CP802	5.33	6.50	1.17	89.33
S4	M	Right	Congenital	CI422	CP802	4.58	5.58	1.00	84.00
S5	M	Left	Congenital	CI422	CP802	5.08	6.08	1.00	84.00
S6	M	Left	Congenital	CI422	CP802	2.83	4.00	1.17	89.33
S7	F	Right	Congenital	CI422	CP802	4.00	5.58	1.58	94.67
S8	F	Left	Congenital	CI422	CP802	5.33	6.33	1.00	84.00
S9	F	Right	Congenital	CI422	CP802	5.17	6.17	1.00	78.67
S10	F	Right	Congenital	CI422	CP802	4.33	5.33	1.00	89.33
S11	M	Right	Congenital	CI422	CP802	4.00	5.00	1.00	78.67
S12	F	Left	Congenital	CI422	CP802	5.5	7.00	1.50	30.67
S13	F	Left	Congenital	CI422	CP802	5.58	6.58	1.00	41.33
S14	M	Right	Congenital	CI422	CP802	5.17	6.17	1.00	25.33
S15	F	Right	Congenital	CI422	CP802	2.87	4.00	1.00	94.67
S16	M	Right	Congenital	CI422	CP802	5.00	6.00	1.00	41.33
S17	M	Left	Congenital	CI422	CP802	4.25	5.25	1.00	46.67
S18	F	Right	Congenital	CI422	CP802	5.25	6.25	1.00	89.33
S19	M	Right	Congenital	CI422	CP802	3.33	4.33	1.00	25.33
S20	M	Right	Congenital	CI422	CP802	5.25	6.25	1.00	84.00
S21	M	Right	Congenital	CI422	CP802	5.25	6.5	1.00	41.33
S22	M	Right	Congenital	CI422	CP802	4.83	6.08	1.25	46.67
S23	F	Left	Congenital	CI422	CP802	4.33	5.33	1.00	73.33
S24	F	Right	Congenital	CI422	CP802	5.33	6.33	1.00	62.67
S25	M	Right	Congenital	CI422	CP802	6.00	7.00	1.00	36.00
S26	M	Right	Congenital	CI422	CP802	4.48	5.58	1.00	78.67
S27	F	Right	Congenital	CI422	CP802	5.58	6.58	1.00	25.33
				Mean		4.78	5.86	1.06	64.64
				Standard deviation		0.82	0.81	0.15	25.58
				Median		5.08	6.08	1.00	78.67

M, male; F, female.

### 2.2 Speech perception testing

Speech perception was assessed using the Picture Identification Test for Kannada-speaking children (Vandana and Yathiraj, 1998). The evaluation involved presenting 25 phonemically balanced bisyllabic words at 45 dB HL through a loudspeaker placed 1 m away at a 45-degree angle towards the implanted ear, using the Piano-Inventis diagnostic audiometer. The child had to listen carefully and identify the words by pointing correct picture out four alternative choices. Initially, child was familiarized with task using trial words. The accurate identification of words was tallied and converted into percentages to determine speech recognition scores. These scores were then adjusted for guessing (Sherbecoe and Studebaker, 2004), resulting in mean speech recognition scores of 64% ± 25.85%.

### 2.3 Ethical consideration

The Institutional Ethics Committee approved the study with approval number No. SH/EC/PhD/AUD-1/2023–24, dated September 22, 2023. Written informed consent was obtained from all the participant’s parents or legal guardians prior to their inclusion in the study.

### 2.4 Stimuli

The charge balanced cathodic leading biphasic pulse train, lasting 36 msec, was utilized to stimulate each electrode. Shorter duration pulse train was presented to avoid the effect of stimulus artifact on the eLLR responses (Gordon et al., 2008). The stimulus was delivered using Custom Sound EP (Version 6) software, a programming pod, and a CP802 speech processor. Each phase in the biphasic pulse train had a width of 37 microseconds, with a 7-microsecond interphase gap, delivered at a rate of 250 pulses per second (pps). The stimulation mode employed was MP1 + 2 (Liebscher et al., 2018), with a stimulus repetition rate of 0.9 pulse trains per second. The biphasic pulse train was applied individually to electrodes located in the apical (E20), middle (E11), and basal (E03) regions. Electrode impedance was measured prior to testing to check for anomalies (open or short circuit) in the stimulating electrode.

The pulse train level was adjusted to achieve the maximum stimulation levels that felt loud yet comfortable. A five-point loudness scale was utilized (very soft, soft, comfortable, loud but okay, very loud and paining) to assess the loudness perception of the pulse train. To establish these levels, the pulse train was initially presented at a starting level of 20 current levels (CL) below the existing comfort levels from the clinical map used by the participant and gradually increased in five CL steps until the child indicated that the sensation was ‘comfortable’ and then increased by two CL until the perception ‘loud but okay’. Children who were unable to express loudness perception through a visual analogue scale were asked to report whether the stimulation was very loud causing any discomfort (He et al., 2016). Furthermore, the researcher monitored the child for any signs of discomfort during the stimulation. The procedure was repeated two times, and the average of the two was considered as the maximum stimulation levels for that electrode. The average stimulation levels for the apical electrode were 209.85 ± 8.87 CL, for the middle electrode were 210.67 ± 8.52 CL, and for the basal electrode were 213.59 ± 8.27 CL.

### 2.5 eLLR recordings

**Figure 1:**
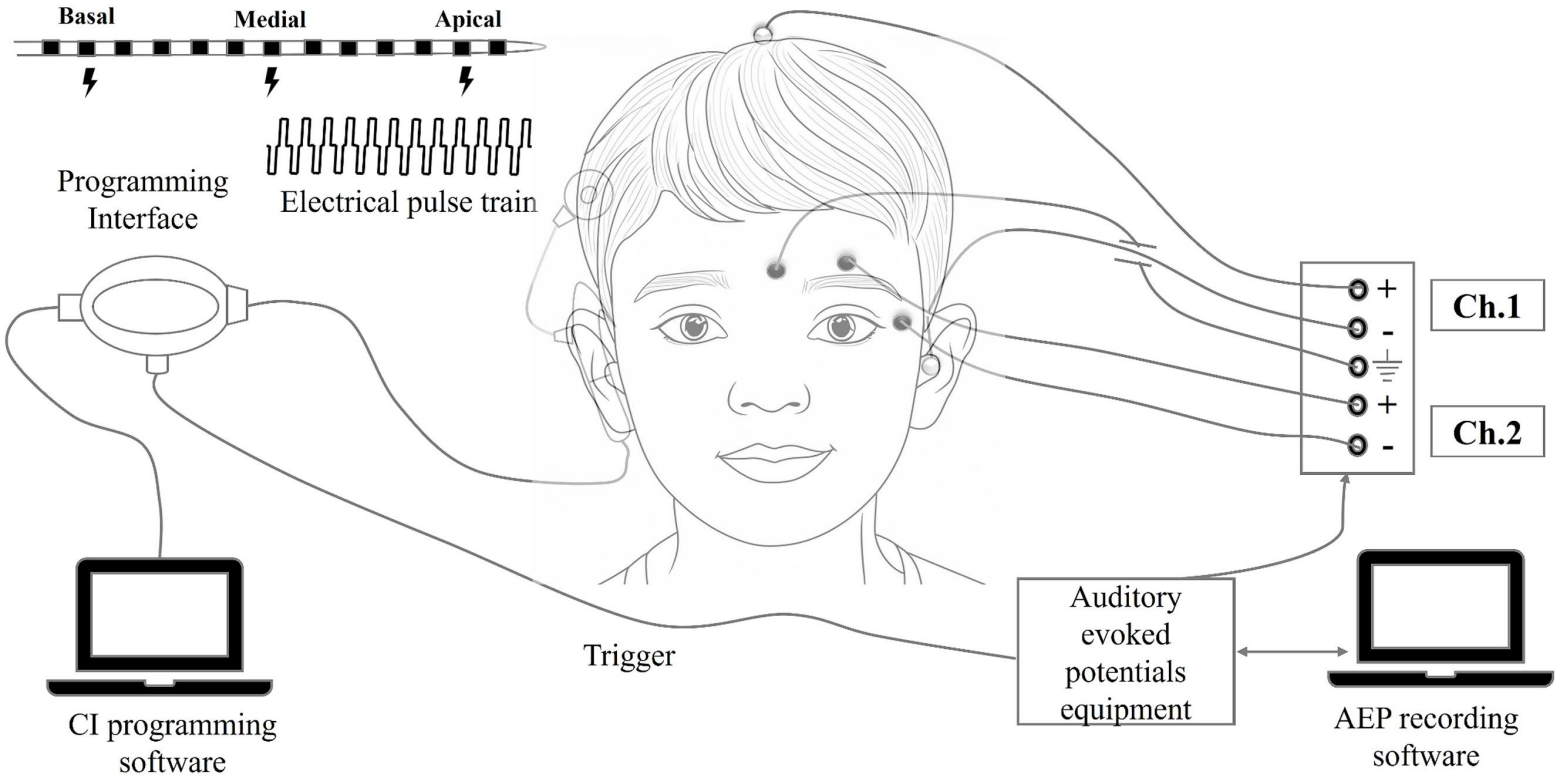
Schematic representation of the equipment setup used during the eLLR recordings.

The eLLR response was captured using the SmartEP auditory evoked potentials (AEP) system (Intelligent Hearing System, Miami, USA). The programming pod was linked to the AEP system through an external trigger cable. During the eLLR recording session, the child was seated comfortably. A surface electrode was positioned at the vertex (Cz) and referenced to an electrode on the opposite mastoid, with a ground electrode on the lower forehead (Fpz). Before electrode placement, a mildly abrasive skin preparation gel was used to ensure that the absolute impedance at each electrode site was below 5 kΩ and the inter-electrode impedance was under 2 kΩ. Responses were recorded at a sampling rate of 1,000 Hz with online bandpass filtering set between 1 and 100 Hz. The presentation of the pulse train was delayed by 320 milliseconds from the trigger onset using Custom Sound EP software to bypass the initial electrical artefact. Subsequently, the recorded response was time-shifted by −320 milliseconds to align with the applied initial delay. The recording consisted of 100 sweeps, with a minimum of two visually replicable responses collected. Two-channel recordings were performed, with one channel dedicated to eLLR recording and the other for monitoring and rejecting eye-blink artifacts. This was accomplished by positioning electrodes in the lateral and superior outer canthi. Epochs with amplitudes exceeding +40 μV or dropping below −40 μV were excluded. A schematic representation of the setup used for eLLR recordings is shown in Figure 1.

### 2.6 Data analysis

eLLR responses were pre-processed using EEGLAB toolbox (Delorme and Makeig, 2004). The eLLR waveform were filtered offline for 1 Hz high pass filtering and 30 Hz low pass filtering with Finite Impulse Response (FIR) filtering. The grand average eLLR responses were plotted using the plot function in the EEGLAB toolbox. The baseline correction was applied to the pre-stimulus interval of −100 to 0 milliseconds using the custom designed MATLAB code (Mathworks, USA). Lin’s concordance correlation coefficient was utilized to evaluate the replicability of the eLLR responses (Polonenko et al., 2023). Recordings with a Lin’s concordance correlation coefficient greater than 0.2 were considered for further analysis. The study assessed various dependent variables, including the latency and absolute amplitude of positive peak P1, P2, and negative peak N1 in milliseconds (msec), and the peak-to-peak amplitudes between P1 and N1 (P1-N1) and between N1 and P2 (N1-P2) in microvolts. The independent variable was the electrode location (apical, middle, and basal). Most commonly the biphasic responses were observed and the eLLR waveforms were visually inspected by two experienced audiologists working with CI children. P1 and N1 peaks were identified as first robust positivity followed by the largest negativity, and P2 peak was identified as the second positive deflection after N1 (Gordon et al., 2005; Sharma et al., 2002a; Wang et al., 2024).

Statistical analyses were conducted using JASP (Jeffreys’s Amazing Statistics Program) version 0.18.3.0 for Windows. The Shapiro–Wilk test was performed to check if variables followed a normal distribution. Parametric statistics were used if the variables were normally distributed; otherwise, non-parametric statistics were utilized. The independent samples *t*-test was utilized to compare eLLR responses between participants implanted in the right ear and those implanted in the left ear across three electrode sites. The Repeated measures one-way ANOVA was employed to compare latency and amplitude measures across the three electrode locations. Post-hoc pairwise comparisons using Bonferroni corrections were conducted to assess the differences across the three electrodes. Additionally, correlation analysis using Pearson or Spearman correlation was utilized to explore the relationship between eLLR measures across electrode locations and the relationship between eLLR measure, speech perception, and age at implantation. A *p*-value <0.05 was considered statistically significant.

## 3 Results

The present study compared the eLLR measured with single electrode stimulation recorded across apical, middle and basal electrodes and assessed the relationship between eLLR measures and speech perception. The Shapiro–Wilk test indicated that the amplitude and latency measures of P1 and N1 followed a normal distribution (*p* > 0.05). However, speech recognition scores and age at implantation were significantly deviant from normal distribution (*p* < 0.05). Of the 27 participants, 19 were implanted in the right ear and 8 in the left ear. An independent samples t-test revealed no significant asymmetry in P1, N1 latency, P1, N1, and P1-N1 amplitudes between those implanted in the right and left ears (*p* > 0.05). Therefore, eLLR recordings of all 27 participants were analysed together across the three electrode sites.

### 3.1 eLLR detection rates

**Figure 2:**
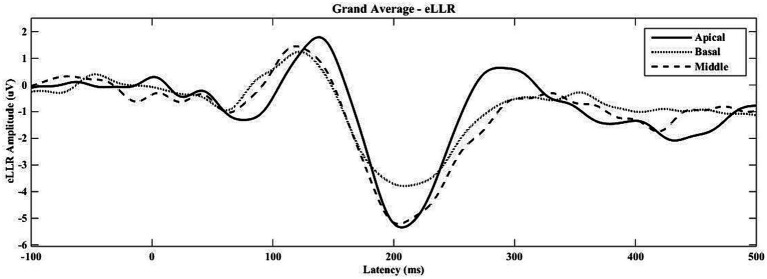
The grand average eLLR responses across the apical (*N* = 27), middle (*N* = 26) and basal (*N* = 24) electrode locations.

**Figure 3:**
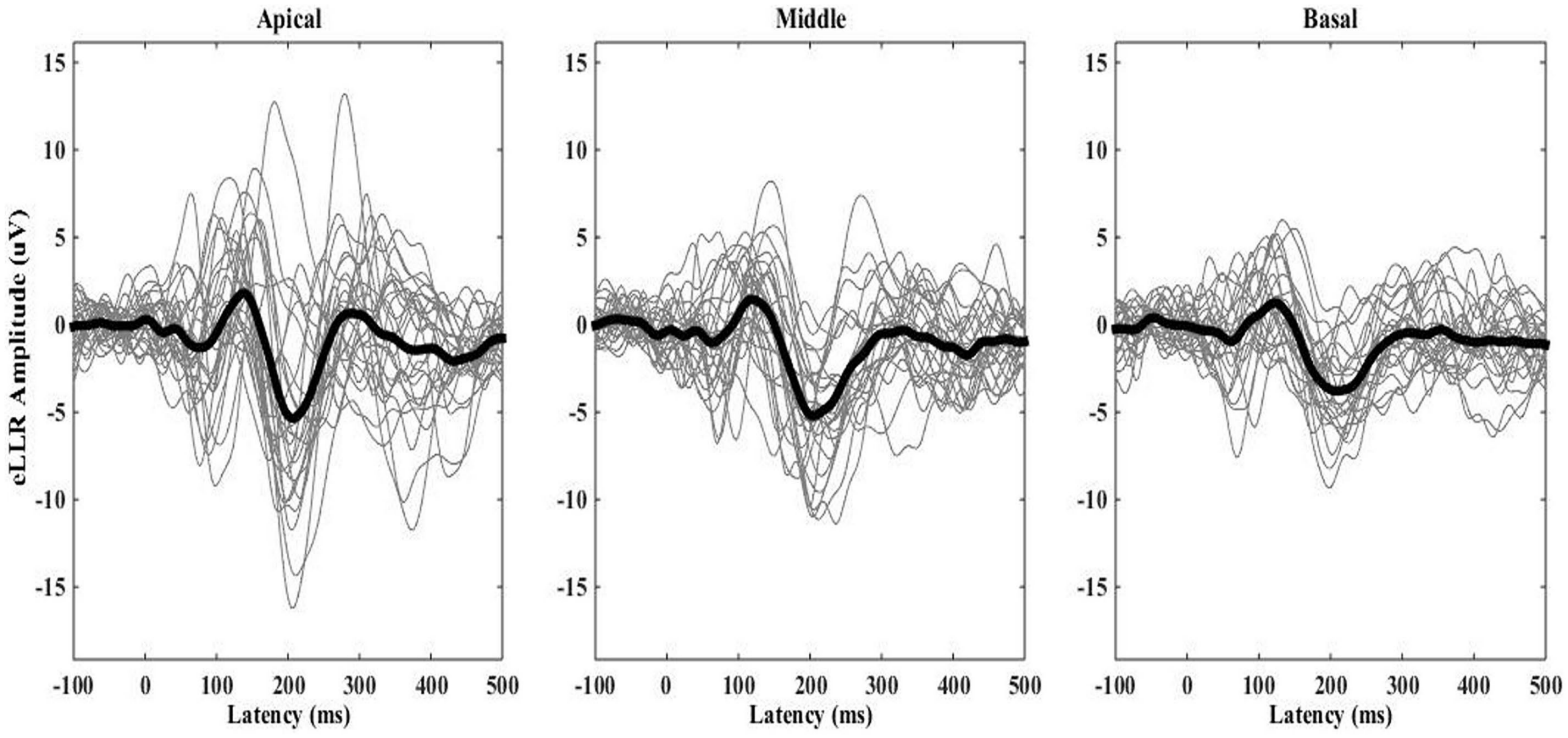
The grand average eLLR responses shown as thick waveform across the apical (*N* = 27), middle (*N* = 26) and basal (*N* = 24) electrode locations. Individual responses are indicated by the thinner waveforms.

eLLRs were present in 77 out of 81 tested electrodes across three electrode locations in 27 participants (27*3 = 81). All participants showed eLLR responses in the apical electrode, whereas S16 had an absent or non-replicable response (Lin’s concordance correlation coefficient ≤ 0.2) in the middle electrode. Similarly, S17, S22, and S27 had absent or non-replicable eLLR responses (Lin’s concordance correlation coefficient ≤ 0.2) in the basal electrode stimulation. Consequently, 27 (100%) eLLR responses from the apical electrode, 26 (96.30%) from the middle electrode, and 24 (88.89%) from the basal electrode stimulation were included for analysis. Figure 2 illustrates the average eLLR responses across the three electrode locations. Figure 3 displays the mean eLLR responses across the three electrode locations, as well as individual participant responses.

The average waveform in Figure 2 reveals a positive peak from 80 to 170 msec, followed by a negative peak from 171 to 270 msec. All 77 eLLR responses analysed displayed P1 and N1 components.

### 3.2 P2 response

**Figure 4:**
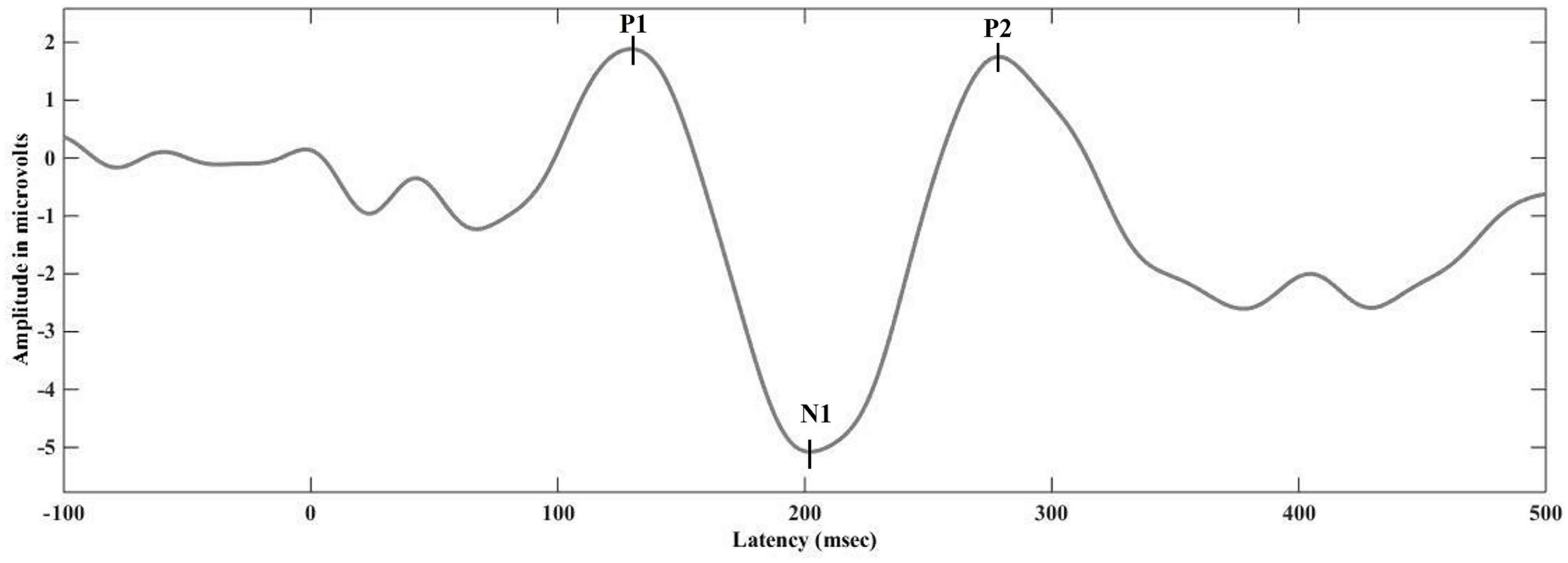
The grand average eLLR responses showing the presence of P2 response (*N* = 13).

Unlike P1 and N1 responses, P2 was not present in all recordings. At the apical electrode, P2 was identifiable in 12 out of 27 responses (44.44%). For middle electrode stimulation, only one eLLR wave (3.85%) showed P2, and none of the basal electrode stimulation revealed a clear P2 response. Figure 4 presents the mean eLLR responses with identifiable P2 peaks across 13 waveforms. The mean P2 latency of 12 eLLR responses with apical electrode simulation was 268.67 ± 41.69 msec with a mean P2 amplitude of 5.02 ± 4.13 μV (N = 12), and the mean N1-P2 amplitude of 13.01 ± 5.51 μV (N = 12). For middle electrode stimulation, P2 was identified in S14, with a latency of 273 msec and P2 amplitude of 7.39 μV and N1-P2 amplitude of 10.80 μV.

### 3.3 Comparison between electrode sites

**Figure 5:**
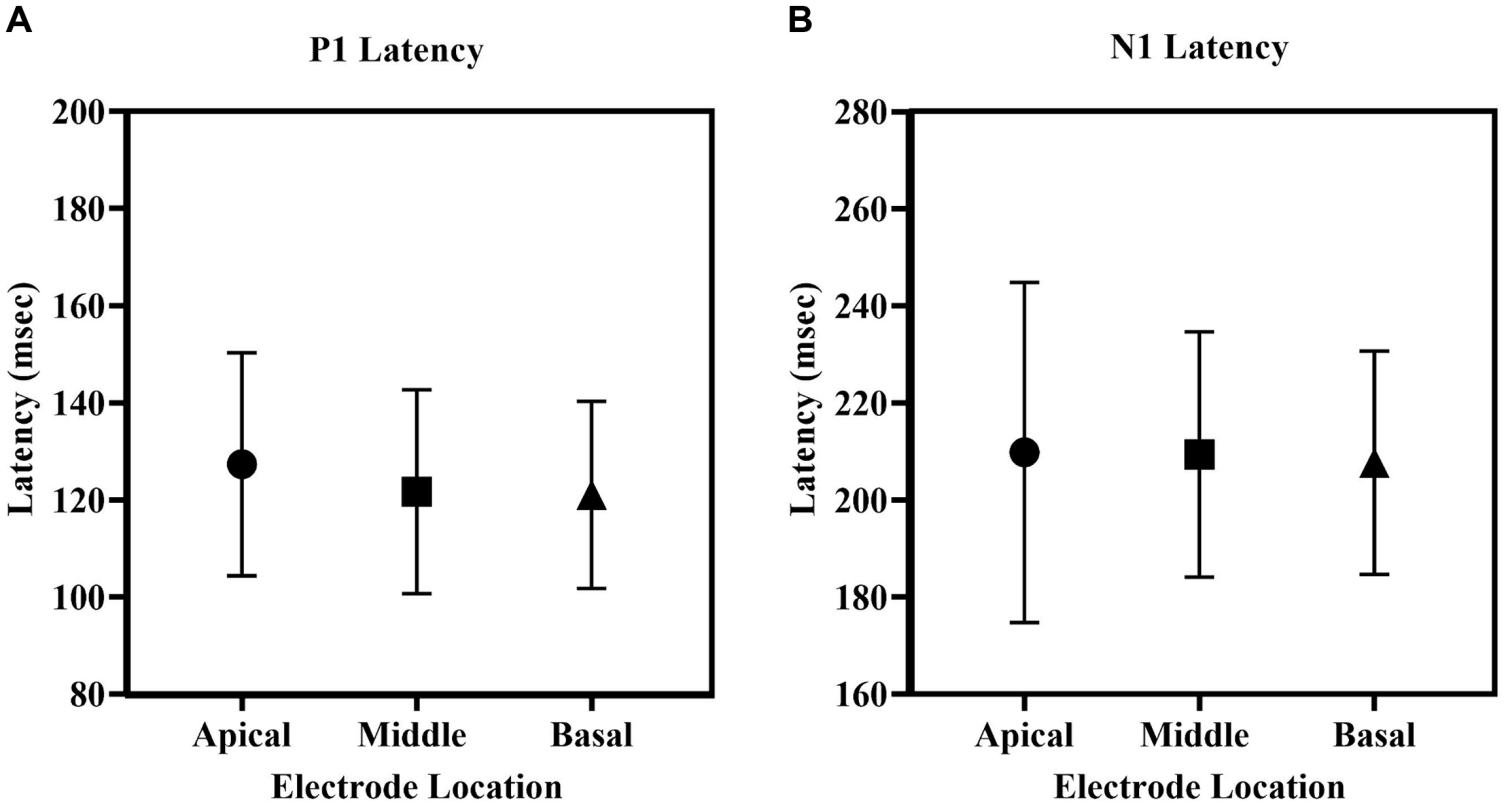
The mean **(A)** P1 latency, **(B)** N1 latency of eLLR responses across apical, middle and basal electrode locations. The error bar indicates one standard deviation from the mean.

Figure 5 depicts the mean latencies for P1 and N1 across the three electrode sites. P1 latency ranged from 76 to 168 msec, and N1 latency ranged from 116 to 289 msec across locations. P1 latency was slightly shorter (~5.5 msec) for middle and basal electrode locations compared to apical electrode stimulation. A repeated-measures one-way ANOVA revealed no significant differences in P1 latency [*F*(2, 44) = 2.485, *p* = 0.095, *η^2^* = 0.101] and N1 latency [*F*(1.56, 34.24) = 0.172, *p* = 0.789, *η^2^* = 0.008] across electrode locations.

**Figure 6:**
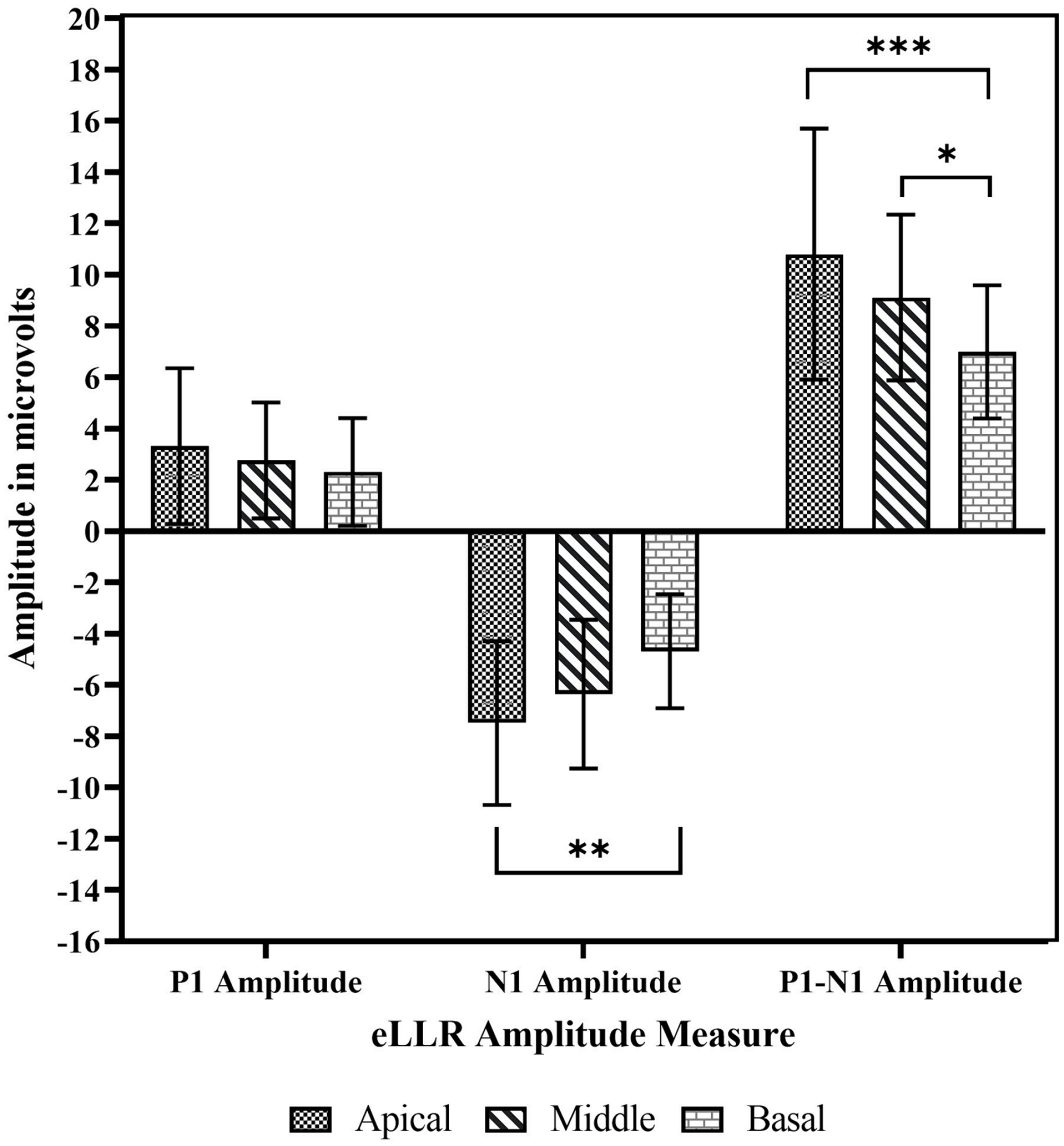
The mean P1 amplitude, N1 amplitude and P1-N1 amplitude across basal, middle apical electrode location. The error bar indicates one standard deviations from the mean. *p < 0.05, **p < 0.01, ***p < 0.001.

Regarding amplitudes, the apical electrode stimulation showed the largest P1 amplitude, followed by the middle, basal electrodes. Similar trends were observed for both the N1 and P1-N1 amplitudes. Figure 6 illustrates the P1 amplitude, N1 amplitude, and P1-N1 amplitude across the three electrode locations. A repeated measures one-way ANOVA indicated no significant difference in P1 amplitude across electrode sites [*F*(2, 44) = 1.903, *p* = 0.161, *η^2^* = 0.080]. However, significant differences in N1 amplitude across locations [*F*(2, 44) = 7.405, *p* = 0.002, *η^2^* = 0.252]. *Post-hoc* comparisons with Bonferroni corrections, revealed a significantly larger N1 amplitude for apical electrode stimulation compared to basal electrode stimulation (*p* = 0.001). Additionally, there were no differences between basal and middle electrode stimulations (*p* = 0.122) or between apical and middle (*p* = 0.271) electrode stimulations. Similar to the N1 amplitude, a statistically significant effect of electrode location was observed in P1-N1 amplitude [*F*(2, 44) = 10.170, *p* < 0.001, *η^2^* = 0.316]. Post-hoc tests revealed a significantly larger P1-N1 amplitude for apical compared to basal stimulation (*p* < 0.001), middle compared to basal stimulation (*p* = 0.082), and no significant differences between apical and middle electrode stimulations (*p* = 0.093).

### 3.4 Correlation analysis

**Figure 7:**
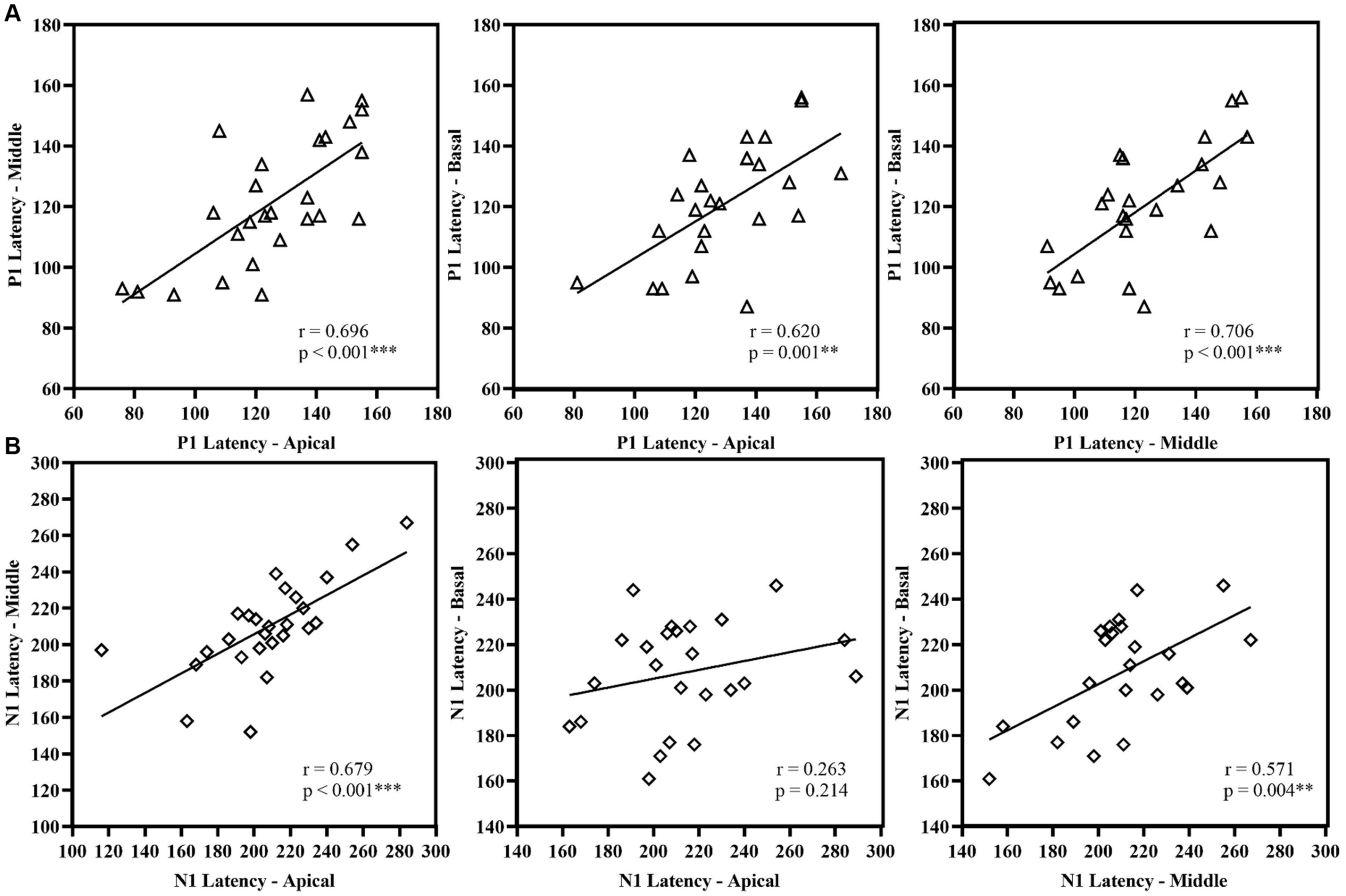
Correlational analysis of latency measures **(A)** P1 Latency and **(B)** N1 Latency across the electrode location. **p < 0.01, ***p < 0.001.

**Figure 8:**
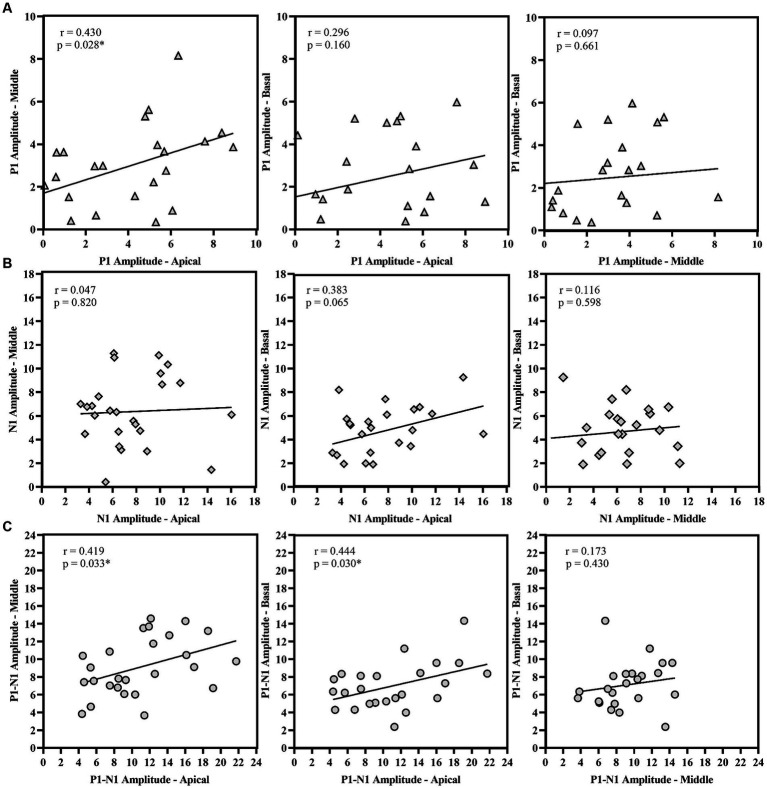
Correlational analysis of amplitude measures **(A)** P1 Amplitude, **(B)** N1 Amplitude, and **(C)** P1-N1 amplitude across the electrode location. *p < 0.05.

P1 latency showed significant correlation across the three electrode locations. N1 latency at the apical electrode significantly correlated with N1 latency at middle electrode; similarly, N1 latency at the middle electrode significantly correlated with N1 latency at basal electrode. However, N1 latency at the apical electrode did not correlate with the response from the basal electrode. For amplitude measures, P1 amplitude and P1-N1 amplitude significantly correlated between the apical and basal electrodes. P1-N1 amplitude also correlated between middle and basal electrodes. N1 amplitude measures did not show significant correlation between electrode locations. Figures 7, 8 display the results of the correlational analysis.

**Figure 9:**
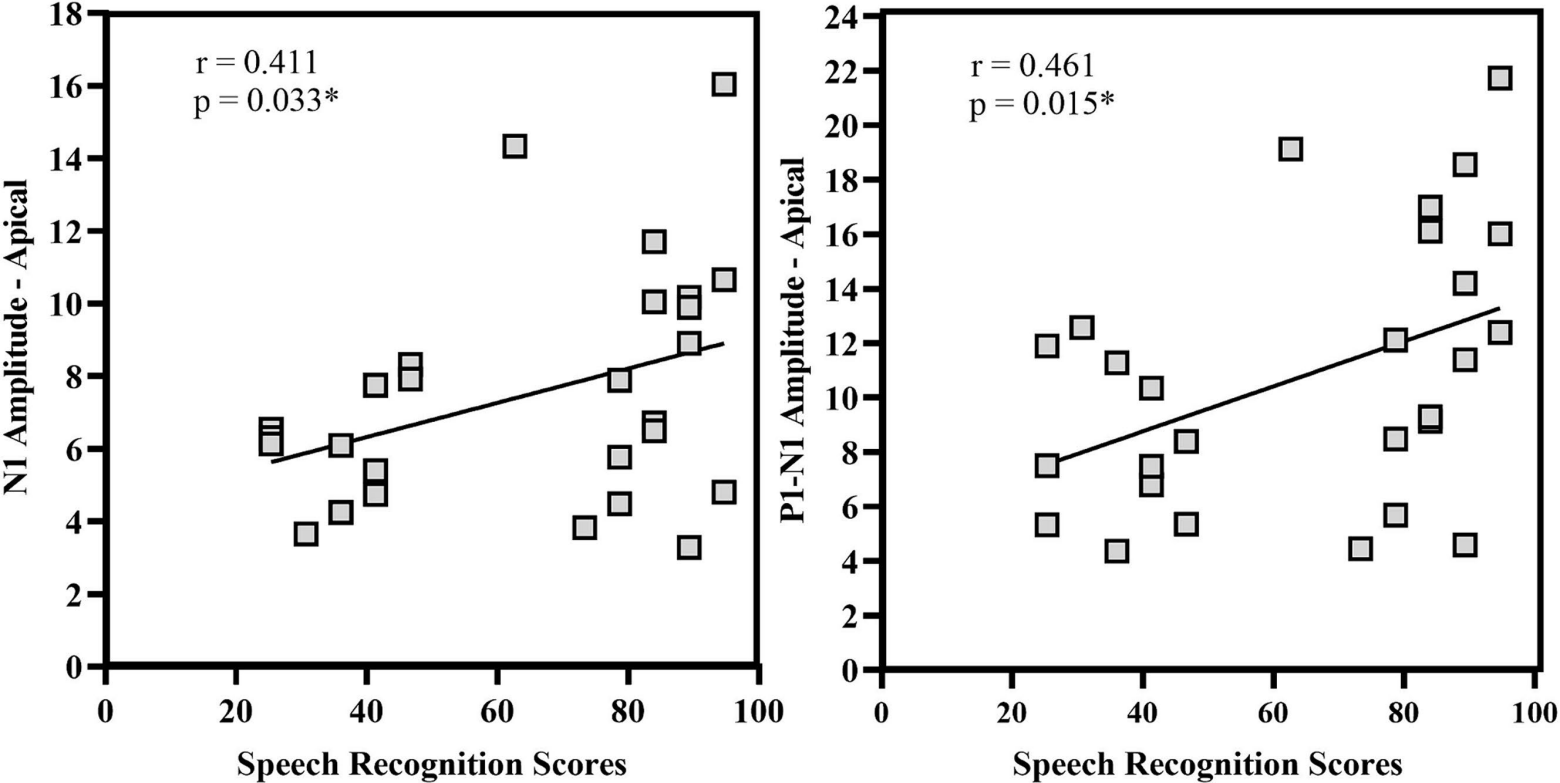
Correlational analysis of N1 amplitude and P1-N1 amplitude for apical electrode stimulation. *p < 0.05.

Spearman’s rho correlation analysis assessed the relationship between speech recognition scores, eLLR measures, and age at implantation. A significant positive correlation between N1 amplitude and P1-N1 amplitude recorded with apical electrode stimulation. Other eLLR measures did not significantly correlate with the speech recognition scores (*p* > 0.05). There was significant negative correlation with age at implantation for N1 absolute amplitude with apical electrode stimulation (rho = −0.384, *p* = 0.048) and P1-N1 peak to peak amplitude with basal electrode stimulation (rho = −0.418, *p* = 0.042). Figure 9 shows the relationship between speech recognition scores and eLLR responses.

## 4 Discussion

The current study compared eLLR responses across three electrode sites (apical, middle, and basal) and investigated the relationship between speech perception and eLLR components. The results revealed a notable difference in eLLR across the electrode sites and moderate correlation between speech perception and eLLR components.

### 4.1 eLLR response detection and morphology

The eLLR response morphology from 77 electrodes across three locations (as shown in Figures 2, 3) was primarily characterized by a positive peak (P1) followed by a negative peak (N1). These findings are consistent with previous research on eLLR responses in children with CI (Gordon et al., 2008; Gordon et al., 2013; Polonenko et al., 2023). Detection rates for eLLR were 100% for apical, 96.30% for middle, and 88.89% for basal electrode stimulation. The higher detection rate for the apical stimulation, as observed in this study, aligns with the adult CI users (Callejón-Leblic et al., 2023; Liebscher et al., 2018; Távora-Vieira et al., 2021; Tavora-Vieira and Ffoulkes, 2023). The mean insertion angle for the CI422 was reported to be 450^o^ (Franke-Trieger and Mürbe, 2015). According to the Greenwood frequency map (Greenwood, 1990), the cochlear duct’s length corresponds to approximately 7.77, 2.86, and 0.85 kHz for E03, E11, and E20, respectively (Dhanasingh and Jolly, 2017; Franke-Trieger and Mürbe, 2015). The lower detection rate in the basal site (E03) may be due to a reduced number of functioning neurons (Liebscher et al., 2018). Távora-Vieira et al. (2022a) reported that adjusting CI map based on the presence of acoustic LLR for four speech tokens (/m/, /t/, /g/, and /s/) significantly improved speech perception in adults. In our study, four participants with absent eLLR responses had speech recognition scores below 50%, suggesting that eLLR with single electrode stimulation could be a valuable tool for optimizing the CI stimulation in children, warranting further validation.

Another notable finding was the presence of a P2 response in 13 out of 77 (16.89%) recordings (shown in the Figure 4), particularly noticeable with apical electrode stimulation. Most studies on LLR in children using CI do not report the details of the P2 response (Polonenko et al., 2023; Sharma et al., 2002a; Sharma et al., 2015a). The origin for N1 and P2 likely originates from different anatomical structures; N1 originates from the posterior auditory cortex, whereas the P2 from the anterior auditory cortex, Broadman’s area 22, and auditory association areas (Ross and Tremblay, 2009; Crowley and Colrain, 2004). In children, the development of the N1 peak bifurcates the positive peak into P1 and P2. The clinical significance of the P2 response warrants further exploration in future studies.

### 4.2 Differences in eLLR across electrode sites

Our results showed that eLLR responses were notably larger with apical electrode stimulation compared to basal stimulation. Specifically, N1 and P1-N1 amplitudes were significantly higher for apical electrode stimulation than for basal stimulation, and P1-N1 responses were notably larger with middle electrode stimulation compared to basal stimulation (Figures 5, 6). This finding is consistent with Visram et al. (2015), who observed significantly lower eLLR amplitudes (N1-P2 amplitude) in the basal region (E03) compared to the middle (E11) and apical region (E20) in adult CI users. No differences in amplitude were observed between the middle and apical electrodes. The decrease in amplitude from the apical to the basal region may be attributed to fewer residual functional auditory neurons and longer auditory deprivation in the basal regions of the cochlea (Liebscher et al., 2018). Another contributing factor could be, eLLR recorded at lower stimulation levels due to the lower tolerance levels for basal electrode stimulation (Visram et al., 2015).

Callejón-Leblic et al. (2023) reported that adult CI users implanted with MED-EL implants showed lower amplitude N1-P2 response in the basal electrode site despite higher stimulation levels. Similarly, larger eLLR response at the apical region could be related to differences in characteristic frequencies at the stimulating electrode sites (Liebscher et al., 2018). The differences were also attributed to the lesser number of neurons and less residual hearing in the basal part compared to apical region (Deniz et al., 2022; Liebscher et al., 2018). In contrast, Kranick et al. (2021) reported similar eLLR responses across the electrode sites. The differences in the findings across studies could be due to the differences in electrode array design (longer vs. shorter length array) (Callejón-Leblic et al., 2023; Kranick et al., 2021; Liebscher et al., 2018; Visram et al., 2015).

Tavora-Vieira and Ffoulkes (2023) found reduced eLLR responses for basal electrode stimulation compared to apical stimulation, along with decreased P1-N1 and N1-P2 amplitudes. Variations in eLLR responses were supported by acoustic LLR with high-frequency stimuli like the /s/ stimulus, which showed absent responses in over half (*N* = 108) of participants and the poorest eLLR morphology for /s/ stimuli (Távora-Vieira et al., 2022a).

Another evidence comes from the differences in electrically evoked compound action potentials (eCAPs) have been observed in CI users (Schvartz-Leyzac and Pfingst, 2016) at the peripheral level. The eCAPs responses displayed higher amplitude when recorded in apical electrodes than in basal electrodes (De Heyning et al., 2016). The increased number of neurons and shorter distance between the electrode and stimulating neurons may have contributed to the higher amplitude in apical electrodes (He et al., 2017). The eLLR response is believed to be influenced by the peripheral response but also reflects variations in central processing of the auditory system (Abbas and Brown, 2015).

Our study suggests differential cortical maturation with reference to different sites of stimulation in the cochlea. The P1 latency was significantly correlated across electrode sites, indicating parallel cortical maturation post-cochlear implantation (Figure 7). However, the N1 amplitude did not show significant correlation across sites and differed notably between apical and basal electrodes (shown in Figure 8). The P1 component is well-established biomarker of cortical maturation in paediatric CI users (Sharma et al., 1997). Additionally, the N1 response plays a crucial role as the neural generators of N1 involve activation of the higher-order auditory cortex (supragranular layers), including connections within and between hemispheres (Eggermont and Ponton, 2003). The N1 response of the eLLR could potentially serve as a measure for higher-order cortical development in children using CI. The maturation of both P1 and N1 responses can indicate the maturation of the primary and higher auditory cortex, respectively (Sharma et al., 2015a). Therefore, the presence of auditory evoked late latency responses confirms the audibility of the sound and availability of sounds for processing by higher-order cortical structures (Korczak et al., 2005). The auditory LLR recorded with speech tokens was used to objectively optimize the stimulation parameters, particularly the upper stimulation levels during CI programming (Távora-Vieira et al., 2022a; Távora-Vieira et al., 2022b). Similarly, the eLLR responses recorded with single electrode stimulation were reported to have potential application to objectively verify the upper stimulation levels (Tavora-Vieira and Ffoulkes, 2023; Deniz et al., 2022).

### 4.3 Relationship between eLLR and speech perception

Our study found a significant positive correlation between N1 amplitude, P1-N1 amplitude, and speech recognition scores (see Figure 9). However, no correlation was observed between eLLR latency measures and speech recognition scores. Various studies have assessed the correlation between acoustic LLR and eLLR both in paediatric and adult populations (Alvarenga et al., 2012; Sharma et al., 2015b; Liebscher et al., 2018; Tavora-Vieira and Ffoulkes, 2023). In adult CI users, Liebscher et al. (2018) identified a positive correlation (*r* = 0.34) between N1 absolute amplitude and N1-P2 peak-to-peak amplitude of electrode 10 (*r* = 0.33) with speech recognition scores. The study also revealed a negative correlation between P2 latency (*r* = −0.35) and speech recognition scores for E19. The association with N1 amplitude may indicate the level of cortical activation. Conversely, the negative correlation with P2 implies enhanced processing speed in individuals with good speech perception (Liebscher et al., 2018).

The emergence of the N1 response indicates the development of pathways connecting the thalamus and cortex, along with connections within the outer layers of the auditory cortex. These pathways play a vital role in transmitting auditory information within the auditory cortex and between different brain hemispheres (Ponton and Eggermont, 2001; Eggermont and Ponton, 2003). In line with the present study, Tavora-Vieira and Ffoulkes (2023) did not report a significant association between eLLR latency measures and speech recognition scores in quiet among adult CI users. However, they did find a notable correlation between P1 (*r* = 0.41) and P2 latency (*r* = −0.49) of all electrodes collectively with speech perception in noise.

Likewise, Cardon and Sharma (2013) identified a significant correlation between P1 latency and Infant Toddler Meaningful Auditory Integration Scale (IT-MAIS) scores in children with ANSD using hearing aids or CI. Nevertheless, the current study did not detect any connection between eLLR latency measures and speech perception abilities. The lack of correlation between eLLR latency measures and speech perception in our study may be due to the restricted age range and duration of CI use among our participants compared to those in other studies. Chronological age and length of CI use can significantly impact P1 latency (Sharma and Dorman, 2006; Gordon et al., 2013).

The current study unveiled a negative correlation between the age at implantation and N1 amplitude of the apical electrode, as well as P1-N1 amplitude in the basal electrode (Figure 9). In Sharma et al. (2015b), reported children implanted at a younger age were more likely to show the N1 component. In contrast, children implanted later exhibited less likelihood of an N1 response. Similar to the P1 response, the N1 response is also affected when children are implanted after the critical period (Sharma et al., 2015b). Delayed maturation of the higher-order cortex (N1 response) may lead to impaired speech perception and a decline in oral language development due to cross-modal reorganization and disconnection of the primary and higher auditory cortex (Sharma et al., 2015a).

### 4.4 Clinical implications

This study indicates that eLLR responses can be elicited using single electrode stimulations in paediatric population. In clinical settings, eLLR responses could be utilized as part of the outcome assessment post-cochlear implantation mapping. The eLLR has the potential to serve as a clinical tool for recording across electrodes to verify appropriate stimulation levels. Evaluating the P1 and N1 components of eLLR could be a valuable approach to assess cortical maturation post-cochlear implantation. The LLR evoked by speech tokens has been reported to exhibit a strong correlation with eLLR using direct single electrode stimulation (Távora-Vieira et al., 2021). The eLLR with single electrode stimulation offers advantages over acoustically evoked LLR (as discussed in introduction) and serve as an alternative tool to optimize upper stimulation during CI programming in both adults (Tavora-Vieira and Ffoulkes, 2023) and children (Deniz et al., 2022). The use of eLLR over other objective measures such as eCAP and electrically evoked stapedial reflex measurements allows for the assessment of cortical function and confirmation of sound perception. Therefore, eLLR could serve as an objective tool to optimize stimulation and enhance outcomes with CI.

### 4.5 Limitations of the study

One potential limitation of the current study is that the eLLR responses with single electrode stimulations were only recorded in three electrodes across the array. A more extensive measurement involving many electrodes across the array would offer insights into cortical activation with multiple CI stimulations. Caution should be exercised when interpreting the relationship between eLLR responses and speech recognition scores, as the eLLR responses were measured with single electrode stimulations, while speech recognition scores were measured with speech stimuli activating multiple electrode sites across the cochlea. However, the cortical activation resulting from stimulation of the three electrode sites tested in the current study would likely impact speech recognition. The apical (E20), middle (E11), and basal electrode (E03) sites encode frequency bands of 438–563, 1813–2063, and 5,313–6,063 Hz, respectively, which could play a significant role in speech perception.

## 5 Conclusion

The current study revealed variations in eLLR components, especially the N1 response, among paediatric CI users, indicating differences in cortical auditory development across frequencies. A significant correlation between N1 amplitude and speech perception could suggest the significance of the maturation of the higher-order auditory cortex in speech perception among children using CI. This study supports using the N1 component of the eLLR as a tool to measure the developmental plasticity of the higher auditory cortex among paediatric CI users. Another noteworthy finding was the identifiable P2 response in the eLLR recorded during apical electrode stimulation. Further studies are needed to explore its clinical significance. In general, the eLLR responses could be readily elicited in the majority of paediatric CI users across different electrode sites. The eLLR testing has potential applications in the paediatric population in evaluating the outcomes with CI.

## Data Availability

The raw data supporting the conclusions of this article will be made available by the authors, without undue reservation.
